# Case of Recovery from Opium

**Published:** 1815-03

**Authors:** Matt. Rowe

**Affiliations:** Member of the Roy. Coll. of Surgeons. 1, *Clarendon-square, Somer's-town.*


### *For the Medical and Physical Journal.*

*Case of Recovery from Opium;* by Mr. M. Rowe.

On the 29th of August, 1814, I was called in to Mrs. S. aged 23, who, determined upon self-destruction, had swallowed a very large portion of liquid laudanum, (according to her subsequent statement, a wine-glass full, containing sixteen drachms).

When I first saw her, the poison had been received into the stomach about half an hour, and she was perfectly insensible, so much so, that she fell off the chair on which she was sitting in a state of utter stupefaction. I immediately dissolved one scruple of antimonium tartarizatum in a pint of water, which was taken at intervals without producing either nausea or vomiting; I, therefore, again dissolved the same quantity in another pint of water, which was also taken in the course of fifteen minutes, and with as little success, for it produced no sensible effect whatever. My patient still remained in a comatose state, totally insensible, excepting at short intervals, when she complained of extreme cold; the pulse so weak that its vibration was scarcely perceptible, and the pupil in some degree dilated. I now dissolved, in two ounces of water, half a drachm of zinci sulphas, adding one drachm of diluted sulphuric acid; all of which I gave at a single dose, and, to my extreme satisfaction, in less than ten minutes it produced a most rapid and favourable change. She became roused from the torpor which had overpowered her; complained of violent sickness, and vomited a large quantity of fluid, in which the laudanum was perceptible, both to taste and smell. I continued for some time to encourage the sickness, by administering frequently chamomile tea, in which was dissolved a few grains of tart. emetic acidulated by sulphuric acid; until, at length, feeling convinced that the poison was totally evacuated, and the sickness having also subsided, I prescribed an aperient, by which the bowels were speedily moved three or four times.

My patient, by this time, was perfectly recovered from the deleterious effects of the opium; but the violent exertion produced by vomiting had, of course, left her weak and debilitated; strengthening medicines soon, however, recovered her from this evil, and, in a few days, I had the satisfaction of seeing her perfectly convalescent.

From an attentive observation of this case, and of the symptoms which were manifested throughout, I am induced to believe, owing to the sedative powers of opium, that, had I had recourse to bleeding (as recommended by Cullen and others) it would not only have considerably retarded, but but might eventually have endangered the recovery of my patient.*

1, *Clarendon-square,* MATT. ROWE,

*Somers'-town.* Member of the Roy. Coll. of Surgeons.

* This short, but very interesting, paper was intended for our Journal before it appeared in a contemporary publication. Besides its importance, we may plead the wish of the writer that it should have the advantage of a more extended circulation, which is our apology for reprinting it.—EDIT.

